# Follicular Lymphoma *In Situ* Presenting as Dermatopathic Lymphadenopathy

**DOI:** 10.1155/2013/481937

**Published:** 2013-12-22

**Authors:** Orah Nnamdi Obumneme, Igbokwe Uche, Banjo Adekunbiola, Akinde Ralph, Irurhe Nicholas

**Affiliations:** ^1^Department of Anatomic and Molecular Pathology, Lagos University Teaching Hospital, P.M.B. 12003, Lagos State, Nigeria; ^2^Department of Cellular Pathology, Queen's Hospital, Rom Valley Way, Romford, UK; ^3^Department of Radiodiagnosis, Lagos University Teaching Hospital, Lagos State, Nigeria

## Abstract

*Introduction*. Follicular lymphoma *in situ* (FLIS) is a recently described entity with few cases recognized worldwide. To our knowledge, this is the first FLIS reported from West Africa. *Case Presentation*. We present the case of a 48-year-old civil servant with axillary lymphadenopathy discovered on routine mammography. On histology, a predominant reactive change with aggregates of melanophages was seen prompting a diagnosis of reactive lymphadenopathy. Immunohistochemistry done in our laboratory showed CD10 and Bcl-6 positive germinal centres with a small population of Bcl-2 positive germinal centre centre cells that were limited to some of the germinal centres. *Conclusion*. This highlights the use of immunohistochemistry in lymph node pathology—a resource which is very limited in our environment.

## 1. Introduction

According to the commentary on the 2008 WHO classification of lymphoid tumors, follicular lymphoma *in situ* (FLIS) refers to lymph nodes with a background of hyperplastic germinal centres harbouring distinct areas with Bcl-2 overexpression in centroblasts and centrocytes [1]. In FLIS, there is a B-cell population with immunophenotypic and genotypic features of follicular lymphoma; however, these B-cells are exclusively localised to germinal centres in morphologically reactive lymph nodes [2].

FLIS was first recognised in 2002 [3]; however, it is not presently clear cut whether this condition is a precursor to full blown follicular lymphoma (FL) [4]. In a series of 34 cases of FLIS identified by Jegalian et al., six had prior or concurrent FL and five had FLIS composite with another lymphoma. Of patients with negative staging at diagnosis and available followup (twenty-one patients), only one developed FL [5].

We present a case recently diagnosed in a middle-aged African lady which to our knowledge is the first reported occurrence in the West African subregion of the world.

## 2. Case Report

A 48-year-old civil servant presented with axillary lymphadenopathy of insidious onset, discovered on routine mammography. The lymph node was excised and sent for histological analysis. She had neither clinically obvious enlarged lymph nodes elsewhere nor a previous history of lymphadenopathy.

Histological examination was done with the aid of immunohistochemistry. The predominant abnormality on H&E examination was a reactive change characterised by follicular hyperplasia, sinus histiocytosis, and expansion of the interfollicular T-cell zones with increased numbers of inter-follicular dendritic cells associated with patchy aggregates of melanophages. These features were considered indicative of a dermatopathic lymphadenopathy by some of the consultant pathologists. However, some others disagreed and felt there was some subtle evidence of lymphoma. This resulted in the use of immunohistochemistry. Immunohistochemistry done in our laboratory showed CD20 positivity in B-cell areas of the lymph node and follicular germinal centres which were CD10 and Bcl-2 positive. Due to our limited immunohistochemistry experience and the few immunohistochemistry panels at our disposal, the blocks were sent to the Department of Cellular Pathology in Queen's Hospital, Rom Valley Way, Romford, Essex, UK.

**Figure 1:**
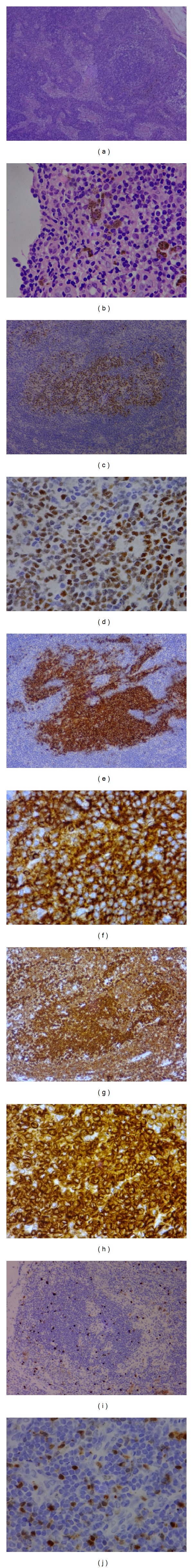
(a and b) H&E showing intact lymph node architecture, sinus histiocytosis, and patchy aggregates of melanophages ((a) ×10, (b) ×40). (c and d): Bcl-6 marking the follicular centre cells ((c) ×10, (d) ×40). (e and f): CD10 marking follicular centre cells ((e) ×10, (f) ×40). (g and h) Bcl-2 staining the cells within the germinal centres strongly ((a) ×10, (b) ×40). (i) Low Ki67 intensity. (j) Patchy S100 staining of interfollicular dendritic cells.

Their analysis revealed secondary follicles with germinal centres that were variably colonised by small CD10 and Bcl-6 positive cells that also overexpressed Bcl-2. The involved follicles had a low proliferation fraction as determined by Ki67 immunohistochemistry. S100 stained the increased numbers of interfollicular dendritic cells (see Figure 1). These features indicated an intrafollicular neoplasia/*in situ* follicular lymphoma.

## 3. Discussion

Follicular lymphoma (FL) is the second most common non Hodgkin lymphoma (NHL) in the Western world [6]. It has an average incidence of 2.6 per 100,000 and median age in the 6th decade [6] and is slightly more common amongst females [7].

In FLIS, the enlarged lymph nodes are usually incidental findings, and the patient has no generalised lymphadenopathy [4]. However, there may be a coexisting FL in the same lymph node or in other nodes as has been reported in a few cases [5, 8]. In the index patient, the lymph nodes were discovered on routine mammography.

In the series of lymph nodes affected by *in situ* follicular lymphoma analysed by Jegalian et al. [5], they found a majority of females (56%) and a peak age of occurrence between the fifth and sixth decades. This report corresponds with the clinical profile of our index patient who is female and aged 48 years.

Follicular lymphoma is a mature B-cell neoplasm thought to be derived from follicular centre B lymphocytes. The lymphoid cells express the immunophenotypic markers associated with germinal centre B-cells, including CD10 and Bcl-6 [9]. In FL, the *Bcl-2* gene on chromosome 18 is merged with the immunoglobulin heavy gene locus on chromosome 14 (t 14:18) (q32;q21) [2]. This results in constitutive activation of the *Bcl-2* gene which is antiapoptotic and leads to accumulation of follicular centre B-cells which may otherwise have died through apoptosis.

Bcl-2 is not expressed in normal follicle centre cells. Inappropriate expression of the *Bcl-2* oncogene has long been believed to be the initial event in malignant transformation to FL [9]. Immunohistochemical staining for Bcl-2 protein also provides a very useful tool for distinguishing between reactive and neoplastic lymphoid follicles as normal germinal centres almost never express Bcl-2 protein [10, 11].

To diagnose FLIS, the following criteria are used [4, 5, 8, 12]: preserved nodal architecture with open sinuses and preserved paracortical regions. On H&E sections, the follicles appear to be reactive. Immunohistochemistry shows follicles positive for CD10, Bcl-6, and CD20. Bcl-2 positive germinal centre cells are confined to the germinal centres of the follicles, do not replace the entire follicle centre, and are not seen in the interfollicular regions or elsewhere in the lymph node. The involved follicles also have a lower proliferation rate with ki67 than the adjacent reactive follicles.

Molecular studies, for example, fluorescence *in situ* hybridization analysis for t(14:18), are only necessary for cases in which immunohistochemistry findings are ambiguous  [12]. In the index case, the immunohistochemistry results were not ambiguous and diagnosis of FLIS was made without FISH.

FLIS has been reported in association with other conditions such as nonlymphoid malignancies and Crohn's disease [12]. In these reports, the FLIS was an incidental finding.

FLIS may transform to follicular lymphoma. In the case series reviewed by Jegalian et al. [5], one out of thirty-four patients reviewed eventually developed follicular lymphoma at the same site. Bonzheim et al. [2] have, however, proposed a clonal evolution from FLIS to manifest follicular lymphoma. This finding has not been supported by other studies with long-term follow-up of patients already diagnosed with FLIS [3, 5].

The index patient, a year after this diagnosis, is presently on followup and has not presented with symptoms or signs of a follicular lymphoma.

*In situ* FL needs to be differentiated from cases of partial involvement of a lymph node by a follicular lymphoma (PFL). In PFL, the architecture of the lymph node is altered when compared to the preserved architecture for FLIS. Also, in FLIS, the germinal centres stain strongly for Bcl-2 and CD10, while in PFL these markers show variable intensity [5]. Other criteria adopted by a panel of experts during the workshop on “early lesions in lymphoid neoplasms” organised by the European Association of Hematopathology in Uppsala, Sweden (2010) include the following: the follicular size is normal in FLIS while being expanded in PFL, the follicles are widely scattered in FLIS while they are grouped together in PFL, the follicular cuff is intact in FLIS while it is attenuated in PFL, and FLIS is composed of almost pure centrocytes while PFL is composed of centrocytes with few centroblasts [13].

## 4. Conclusion

This report highlights a hitherto unreported entity in the West African subregion of the world and highlights the need for immunohistochemistry in the diagnosis of lymph node pathology—a resource which is very limited in this environment.

## Learning Points

1. FLIS is a rare entity.
2. This is the first case reported in this subregion.
3. Immunohistochemistry is very relevant in lymph node pathology—a resource which is scarcely available in this environment.
